# HUMeral Shaft Fractures: MEasuring Recovery after Operative versus Non-operative Treatment (HUMMER): a multicenter comparative observational study

**DOI:** 10.1186/1471-2474-15-39

**Published:** 2014-02-11

**Authors:** Kiran C Mahabier, Esther MM Van Lieshout, Hugo W Bolhuis, P Koen Bos, Maarten WGA Bronkhorst, Milko MM Bruijninckx, Jeroen De Haan, Axel R Deenik, Boudewijn J Dwars, Martin G Eversdijk, J Carel Goslings, Robert Haverlag, Martin J Heetveld, Albert JH Kerver, Karel A Kolkman, Peter A Leenhouts, Sven AG Meylaerts, Ron Onstenk, Martijn Poeze, Rudolf W Poolman, Bas J Punt, W Herbert Roerdink, Gert R Roukema, Jan Bernard Sintenie, Nicolaj MR Soesman, Andras KF Tanka, Edgar JT Ten Holder, Maarten Van der Elst, Frank HWM Van der Heijden, Frits M Van der Linden, Peer Van der Zwaal, Jan P Van Dijk, Hans-Peter W Van Jonbergen, Egbert JMM Verleisdonk, Jos PAM Vroemen, Marco Waleboer, Philippe Wittich, Wietse P Zuidema, Suzanne Polinder, Michael HJ Verhofstad, Dennis Den Hartog

**Affiliations:** 1 Trauma Research Unit Department of Surgery, Erasmus MC, University Medical Center Rotterdam, P.O. Box 2040, 3000 CA Rotterdam, The Netherlands; 2 Department of Surgery, Gelre Hospital, P.O. Box 9014, 7300 DS Apeldoorn, The Netherlands; 3 Department of Orthopaedic Surgery, Erasmus MC, University Medical Center Rotterdam, P.O. Box 2040, 3000 CA Rotterdam, The Netherlands; 4 Department of Surgery, Bronovo Hospital, P.O. Box 96900, 2509 JH The Hague, The Netherlands; 5 Department of Surgery, IJsselland Hospital, P.O. Box 690, 2900 AR Capelle a/d IJssel, The Netherlands; 6 Department of Surgery, Westfriesgasthuis, P.O. Box 600, 1620 AR Hoorn, The Netherlands; 7 Department of Orthopaedic Surgery, Bronovo Hospital, P.O. Box 96900, 2509 JH The Hague, The Netherlands; 8 Department of Surgery, Slotervaart Hospital, P.O. Box 90440, 1006 BK Amsterdam, The Netherlands; 9 Department of Surgery, St. Jansdal Hospital, P.O. Box 138, 3840 AC Harderwijk, The Netherlands; 10 Trauma Unit Department of Surgery, Academic Medical Center, P.O. Box 22660, 1100 DD Amsterdam, The Netherlands; 11 Department of Surgery, Onze Lieve Vrouwe Gasthuis, P.O. Box 95500, 1090 HM Amsterdam, The Netherlands; 12 Department of Surgery, Kennemer Gasthuis, P.O. Box 417, 2000 AK Haarlem, The Netherlands; 13 Department of Surgery, Sint Franciscus Gasthuis, P.O. Box 10900, 3004 BA Rotterdam, The Netherlands; 14 Department of Surgery, Rijnstate, P.O. Box 9555, 6800 TA Arnhem, The Netherlands; 15 Department of Surgery, Zaans Medical Center, P.O. Box 210, 1500 EE Zaandam, The Netherlands; 16 Department of Surgery, Medical Center Haaglanden, P.O. Box 432, 2501 CK The Hague, The Netherlands; 17 Department of Orthopaedic Surgery, Groene Hart Ziekenhuis, P.O. Box 1098, 2800 BB Gouda, The Netherlands; 18 Department of Trauma Surgery, Maastricht University Medical Center, PO Box 5800, 6202 AZ Maastricht, The Netherlands; 19 Department of Orthopaedic Surgery, Onze Lieve Vrouwe Gasthuis, P.O. Box 95500, 1090 HM Amsterdam, The Netherlands; 20 Department of Surgery, Albert Schweitzer Hospital, P.O. Box 444, 3300 AK Dordrecht, The Netherlands; 21 Department of Surgery, Deventer Hospital, P.O. Box 5001, 7400 GC Deventer, The Netherlands; 22 Department of Surgery, Maasstad Hospital, P.O. Box 9100, 3007 AC Rotterdam, The Netherlands; 23 Department of Surgery, Elkerliek Hospital, P.O. Box 98, 5700 AB Helmond, The Netherlands; 24 Department of Surgery, Vlietland Hospital, P.O. Box 215, 3100 AE Schiedam, The Netherlands; 25 Department of Surgery, Spaarne Hospital, P.O. Box 770, 2130 AT Hoofddorp, The Netherlands; 26 Department of Orthopaedic Surgery, IJsselland Hospital, P.O. Box 690, 2900 AR Capelle a/d IJssel, The Netherlands; 27 Department of Surgery, Reinier de Graaf Gasthuis, P.O. Box 5011, 2600 GA Delft, The Netherlands; 28 Department of Surgery, St Elisabeth Hospital, P.O. Box 90151, 5000 LC Tilburg, The Netherlands; 29 Department of Surgery, Groene Hart Ziekenhuis, P.O. Box 1098, 2800 BB Gouda, The Netherlands; 30 Department of Orthopaedic Surgery, Medical Center Haaglanden, P.O. Box 432, 2501 CK The Hague, The Netherlands; 31 Department of Surgery, Hospital Gelderse Vallei, P.O. Box 9025, 6710 HN Ede, The Netherlands; 32 Department of Orthopaedic Surgery, Deventer Hospital, P.O. Box 5001, 7400 GC Deventer, The Netherlands; 33 Department of Surgery, Diakonessenhuis, P.O. Box 80250, 3508 TG Utrecht, The Netherlands; 34 Department of Surgery, Amphia Hospital, P.O. Box 90158, 4800 RK Breda, The Netherlands; 35 Department of Surgery, Admiraal de Ruyter Hospital, P.O. Box 106, 4460 BB Goes, The Netherlands; 36 Department of Surgery, St. Antonius Hospital, P.O. Box 2500, 3430 EM Nieuwegein, The Netherlands; 37 Department of Trauma Surgery, VU University Medical Center, P.O. Box 7057, 1007 MB Amsterdam, The Netherlands; 38 Department of Public Health, Erasmus MC, University Medical Center Rotterdam, P.O. Box 2040, 3000 CA Rotterdam, The Netherlands

## Abstract

**Background:**

Fractures of the humeral shaft are associated with a profound temporary (and in the elderly sometimes even permanent) impairment of independence and quality of life. These fractures can be treated operatively or non-operatively, but the optimal tailored treatment is an unresolved problem. As no high-quality comparative randomized or observational studies are available, a recent Cochrane review concluded there is no evidence of sufficient scientific quality available to inform the decision to operate or not. Since randomized controlled trials for this injury have shown feasibility issues, this study is designed to provide the best achievable evidence to answer this unresolved problem. The primary aim of this study is to evaluate functional recovery after operative versus non-operative treatment in adult patients who sustained a humeral shaft fracture. Secondary aims include the effect of treatment on pain, complications, generic health-related quality of life, time to resumption of activities of daily living and work, and cost-effectiveness. The main hypothesis is that operative treatment will result in faster recovery.

**Methods/design:**

The design of the study will be a multicenter prospective observational study of 400 patients who have sustained a humeral shaft fracture, AO type 12A or 12B. Treatment decision (*i.e.*, operative or non-operative) will be left to the discretion of the treating surgeon. Critical elements of treatment will be registered and outcome will be monitored at regular intervals over the subsequent 12 months. The primary outcome measure is the Disabilities of the Arm, Shoulder, and Hand score. Secondary outcome measures are the Constant score, pain level at both sides, range of motion of the elbow and shoulder joint at both sides, radiographic healing, rate of complications and (secondary) interventions, health-related quality of life (Short-Form 36 and EuroQol-5D), time to resumption of ADL/work, and cost-effectiveness. Data will be analyzed using univariate and multivariable analyses (including mixed effects regression analysis). The cost-effectiveness analysis will be performed from a societal perspective.

**Discussion:**

Successful completion of this trial will provide evidence on the effectiveness of operative versus non-operative treatment of patients with a humeral shaft fracture.

**Trial registration:**

The trial is registered at the Netherlands Trial Register (NTR3617).

## Background

Humeral shaft fractures are associated with a profound temporary (in elderly sometimes even permanent) impairment of independence and quality of life. Fractures of the humeral shaft account for 1-3% of all fractures [1]. The cumulative incidence shows a peak in the working population (14.5/100,000 person years) as well as in the elderly (60/100,000) [1-3].

Humeral shaft fractures can be treated operatively or non-operatively. Operative treatment is mostly performed by intramedullary nailing, plate osteosynthesis, or external fixation. Non-operative immobilization is mostly done with a functional (Sarmiento) brace [4]. Operative and non-operative treatment strategies both have their pros and cons. Operative fracture fixation allows for early mobilization, which may lead to earlier functional recovery and reduced pain. However, surgical complications and fixation failure may occur [5]. Non-operative treatment may be associated with more pain (as the fracture is not stabilized) and discomfort (due to pain and immobilization) in the first weeks and may be associated with a higher malunion risk due to the lack of fracture re-alignment [6,7]. Longer immobilization may delay functional recovery.

Complications of operative and non-operative treatment overlap and data are lacking to determine treatment relatedness. Non-union occurs in 15-30% after operative treatment [5] versus 2-23% after non-operative treatment (for which most patients require secondary surgical treatment) [6,7]. The most feared disabling complication is radial nerve palsy, occurring in 2-17% of all patients [8-10]. A systematic review (n = 4,517 patients) reported an average radial nerve palsy of 11.8%. Although 70% recover spontaneously, the palsy was permanent in 12% of cases accounting for a substantial impairment and costs [9].

Regaining function is extremely important from a patient and societal perspective. From the few retrospective and prospective case series published, each using other outcomes, better functional outcome is expected after operative treatment [8,11-17].

The best type of treatment is still debated. Surgeons state that their experience, patient characteristics and expected physical demands in daily living guides treatment decision. In the elderly patients, some surgeons might prefer immobilization while others may primarily operate as they fear inferior functional outcome after non-operative treatment. In younger patients, some surgeons directly perform a surgical intervention while others primarily choose non-operative immobilization, followed by surgical intervention if needed. However, our retrospective study showed an approximately 50% operation rate irrespective of fracture subclass with no obvious differences in patient or fracture characteristics across classes [18]. Since randomized or high-quality comparative observational studies are lacking, a recent Cochrane review concluded there is no evidence of sufficient scientific quality available to inform the decision to operate or not [19]. High-quality clinical studies are thus urgently needed to resolve this clinically relevant problem. RCTs for this injury have shown feasibility issues; one RCT continued as an observational study due to severe recruitment problems [20]. The HUMMER study is designed to provide the best achievable evidence to answer this unresolved problem using an observational trial design.

The primary objective of this study is to examine the effect of operative versus non-operative treatment on the DASH (Disabilities of the Arm, Shoulder, and Hand) score, reflecting functional outcome and pain of the upper extremity, in adult patients who sustained a humeral shaft fracture. Secondary aims are to examine the effect of operative versus non-operative treatment on functional outcome, the level of pain, range of motion of the shoulder and elbow joint, the rate of secondary interventions and complications, the time to resumption of work and activities of daily living, health-related quality of life, costs, and cost-effectiveness in these patients.

## Methods/design

### Study design

The HUMMER trial will follow a multicenter, prospective observational trial design. Approximately 30 hospitals in The Netherlands will participate.

The decision to provide operative or non-operative treatment will be left to the discretion of the attending physician. We chose an observational design because a randomized controlled trial (RCT) would currently not be feasible. Many surgeons prefer not to participate in trials that involve randomization [21] and we know from experience that patients easily refuse to be randomized between operative and non-operative treatment. Inclusion problems were the main reason for failure of a previous RCT with the same research question as this study [20]. Well-designed and adequately reported observational studies are good alternatives to RCTs [22,23]. Preference of observational studies over RCTs in orthopedic trauma has been acknowledged [24,25]. They lead to similar outcomes without the limitations of randomization which may in practice decrease the validity of the outcomes [26,27]. These designs are increasingly used and accepted in surgical studies [28]. In order to answer our research question, we will make adjustments in the statistical analysis by using the propensity matching score method [29-32].

The trial is registered at the Netherlands Trial Register (NTR3617).

### Recruitment and consent

Eligible persons presenting to the ED with a humeral shaft fracture will be informed about the trial at the ED. After an explanation of the study, they will receive information and a consent form from the attending physician, the clinical investigator, or a research assistant. Patients meeting all inclusion criteria and none of the exclusion criteria will be included while they are still at the ED or at the time of their first outpatient visit.

As with many surgical trials, patients and surgeons cannot be blinded for treatment. In order to reduce bias, a research physician or research assistant will perform the follow-up measurements using a standardized protocol. Radiographs can also not be blinded for treatment; however, evaluating radiographs in duplicate by two trauma surgeons independently will improve reliability of fracture healing assessment. In case of disagreement they will discuss the results until they reach consensus. Finally, the analysis will be performed by a statistician without knowledge of treatment.

### Study population

All persons aged 18 years or older presenting to the ED with a humeral shaft fracture (AO type 12A or 12B) are eligible for inclusion [33]. The AO type 12C fractures will be excluded due to their low occurrence rate. Humeral shaft fractures are defined as fractures located in the area between the surgical neck and the area immediately above the supracondylar ridge.

Patients meeting the following inclusion criteria are eligible for enrolment:

1. Adult men or women aged 18 years or older (with no upper age limit)

2. A fracture of the humeral shaft, AO class 12A or 12B (confirmed on X-ray)

3. Operation within 14 days after presentation to the ED (if this is the treatment of choice)

4. Provision of informed consent by patient

If any of the following criteria applies, patients will be excluded:

1. Patients with concomitant injuries affecting treatment and rehabilitation of the affected arm

2. Patients with a humeral fracture treated with an external fixator

3. Patients with a pathological, recurrent or open humeral shaft fracture

4. Patients with neurovascular injuries requiring immediate surgery (excl. radial nerve palsy)

5. Additional traumatic injuries of the affected arm that influence upper extremity function

6. Patients with an impaired upper extremity function (*i.e.*, stiff or painful arm or neurological disorder of the upper limb) prior to the injury

7. Retained hardware around the affected humerus

8. Patients with rheumatoid arthritis

9. Bone disorder which may impair bone healing (excluding osteoporosis)

10. Patients incapable of ensuring follow-up (*e.g.*, no fixed address or cognitive impairment)

11. Insufficient comprehension of the Dutch language to understand the rehabilitation program and other treatment information, as judged by the treating physician of researcher

Exclusion of a patient because of enrolment in another ongoing drug or surgical intervention trial will be left to the discretion of the attending surgeon on a case-by-case basis.

### Intervention

The decision on treatment will be left to the discretion of the attending surgeon. The choice will be between operative and non-operative treatment. Also, the rehabilitation after treatment will not be standardized, but will be provided as in real life. Although this may create some heterogeneity across groups, it will improve the generalizability of the study results.

If a surgeon decides to operate the patient, the choice between plate osteosynthesis or intramedullary nailing will be left to the treating surgeon. No restrictions will be applied to the approach for reduction and fixation of the fracture, *e.g.*, open or closed, antegrade or retrograde. The type and brand of the materials as well as the use of cerclage wires and other elements of the surgery will be left to the surgeon, local availability and expertise. Critical elements of the operative treatment will be recorded (*e.g.*, type of implant, identification of the radial nerve, surgical approach, operative delay, duration of surgery) and the effect on outcome will be assessed.

In order to maximize generalizability, the type of non-operative treatment will also be left to the attending surgeon. Usually it consists of a splint, plaster, collar and cuff or hanging cast for 1–2 weeks, followed by a Sarmiento brace for 4–6 weeks. Critical elements of this treatment will be recorded and the effect on outcome will be assessed.

Due to a lack of evidence favoring a particular approach, the physical therapy and rehabilitation program will be recorded but not standardized. This will improve generalization of the study results.

### Outcome measures

The Disabilities of the Arm, Shoulder and Hand (DASH) outcome measure will serve as primary outcome measure. The DASH is a validated 30-item, self-report questionnaire designed to help describe the disability experienced by people with upper-limb disorders and also to monitor changes in symptoms and function over time [34,35]. It is scored in two components: the disability/symptom section (30 items, scored 1–5) and two optional Work and high performance Sport/Music modules (each 4 items, scored 1–5). The DASH disability/symptom score is a summation of the responses to 30 questions on a scale of 1 to 5, with an overall score ranging from 0 (no disability) to 100 (severe disability). At least 27 of the 30 items must be completed for a score to be calculated. The DASH optional modules aim to measure symptoms and function in athletes, performing artists and other workers whose jobs require a high degree of physical performance. These optional models are scored separately and each contains four items, scored 1–5. All items must be completed for a score to be calculated.

The secondary outcome measures are:

• Constant score

• Pain level at both sides (VAS)

• Range of Motion of the shoulder and elbow joint at both sides

• Rate of complications

• Rate of secondary interventions

• Time to resumption of work and other activities of daily living

• Health-related quality of life: SF-36 and EQ-5D

• Radiographic healing

• Cost of health care use and production loss

• Cost-effectiveness

The Constant score reflects both function and pain [36]. This scoring system consists of four variables that are used for assessing shoulder function. The right and left shoulder are assessed separately. The subjective variables are pain (15 points), activities of daily living (ADL; *i.e.*, sleep, work, recreation/sport; 10 points), and arm positioning (10 points), which give a total of 35 points. The objective variables are range of motion (ROM; 40 points) and strength (25 points), which give a total of 65 points. ROM includes forward flexion (10 points), lateral elevation (10 points), external rotation related to the head (10 points) and internal rotation related to the spine column (10 points). ROM will be measured with a goniometer. Strength of abduction will be measured using a calibrated spring balance.

Pain level will be determined using a 10-point Visual Analog Scale (VAS), in which 0 implies no pain and 10 implies the worst possible pain.

The range of motion (ROM) of the shoulder (*i.e.*, abduction and forward flexion) and the elbow joint (*i.e.*, flexion and extension) will be measured using a goniometer. Both sides will be assessed separately, and the loss of ROM will be calculated.

Complications will be recorded from medical charts. Complications may include: 1) surgical site infection; 2) wound dehiscence; 3) skin problems (*e.g.*, skin at risk, skin necrosis); 4) dystrophia; 5) radial nerve palsy; 6) malunion; 7) implant failure (screw breakout); 8) cuff pathology; 9) secondary fracture dislocation; or 10) non-union. Non-union is defined as a failure to heal at twenty-six weeks post fracture with no progress towards healing seen on the most recent radiographs [37].

Secondary intervention within one year of initial treatment to promote fracture healing, relieve pain, treat infection, or improve function will be recorded from medical charts. Interventions will be categorized as: 1) osteosynthesis with or without bone grafting; 2) implant exchange with or without bone grafting; 3) implant removal; 4) incision and drainage for superficial surgical site infection; or 5) incision and drainage for deep surgical site infection. The indication and admission duration for all intervention will also be recorded.

Presence of radiographic healing will be determined using X-rays. Fracture consolidation is defined when one of the three criteria listed is present; 1) bridging of fracture by callus/bone trabeculae or osseous bone; 2) obliteration of fracture line/cortical continuity; or 3) bridging of fracture at three out of four cortices.

The time to resumption of work and activities of daily living will be recorded using a custom-made questionnaire.

The Short-Form 36 (SF-36) is a validated multi-purpose, health survey with 36 questions, representing eight health domains that are combined into a physical and a mental component scale [38]. The Physical Component Summary (PCS) combines the health domains physical functioning (PF; 10 items), role limitations due to physical health (RP; 4 items), bodily pain (BP; 2 items), and general health perceptions (GH; 5 items). The Mental Component Summary (MCS) combines the health domains vitality, energy, or fatigue (VT; 4 items), social functioning (SF; 2 items), role limitations due to emotional problems (RE; 3 items), and general mental health (MH; 5 items). Scores ranging from 0 to 100 points are derived for each domain, with lower scores indicating poorer function. These scores will be converted to a norm-based score and compared with the norms for the general population of the United States (1998), in which each scale was scored to have the same average (50 points) and the same standard deviation (10 points).

The EuroQol-5D is a validated questionnaire for measuring health-related quality of life [39,40]. Its use is recommended for assessing quality of life in trauma patients, especially for economic assessments [41,42]. The EQ-5D descriptive system consists of five dimensions of health (mobility, self-care, usual activities, pain/discomfort and anxiety/depression). Scores are converted to a utility score ranging from zero to one, with lower scores indicating poorer quality of life. The EQ VAS records the respondents self-rated health status on a vertical (0–100) visual analog scale.

The cost-effectiveness analysis will be performed from a societal perspective and will include costs for health care and production losses. Patients will be asked to complete a custom-made questionnaire that contains detailed information on both items. Health care costs will include general practice care, medical specialist care, nursing care, physical therapy, hospitalization, medication, home care, and other costs directly associated with diagnosis, treatment, and rehabilitation.

In addition to the outcome variables mentioned above, the following data will be collected:

a) Intrinsic variables (baseline data): age, gender, American Society of Anesthesiologists’ ASA classification, tobacco consumption, alcohol consumption, comorbidities (including osteoporosis), dominant side, and medication use.

b) Injury related variables: affected side, mechanism of injury, fracture classification according to the AO classification system, additional injuries, and admission duration.

c) Intervention-related variables: time between injury and start of treatment, days of collar and cuff, sling or plaster, time between injury and start of physical therapy, and number of physical therapy sessions.

### Study procedures

Clinical evaluation will occur at two weeks (7–21 days window), six weeks (4–8 weeks window), three months (11–15 weeks window), six months (6–7 months window), and 12 months (12–14 months window) after start of treatment [Table 1]. These visits are standard of care for the targeted patient group. At each follow-up visit, the research coordinator or research assistant will ascertain patient status (*i.e.*, adverse events/complications, secondary interventions, etcetera, and will verify information within medical records).

**Table 1:** Schedule of events

**Radiographs & events**	**Screening**	**Enrolment**	**Baseline**	**Post**	**2 weeks**	**6 weeks**	**3 months**	**6 months**	**12 months**
				**surgery**	**(7–21 d)**	**(4–8 we)**	**(11–15 we)**	**(6–7 mo)**	**12-14 mo)**
X-ray	X						X^1^	X^1^	X^1^
Screening	X								
Informed consent		X							
Baseline data			X						
Surgical report form				X					
DASH					X	X	X	X	X
Pain (VAS)					X	X	X	X	X
SF-36					X	X	X	X	X
EQ-5D					X	X	X	X	X
Clinic FU					X	X	X	X	X
Range of motion					X	X	X	X	X
Secondary interventions					X	X	X	X	X
Complications					X	X	X	X	X
Health care consumption					X	X	X	X	X
ADL/work resumption					X	X	X	X	X
Physical therapy					X	X	X	X	X
Constant score						X	X	X	X
Early withdrawal					*	*	*	*	*

^1^X-rays will be taken according to local protocol; all X-rays after three months will be analyzed. The six-month X-ray is needed for assessing fracture healing. If no signs of healing are seen at six months, the 12-month X-ray is also required.

*Only at time of withdrawal.

At each follow-up visit, the range of motion of the shoulder and elbow will be measured using a goniometer by a physician or research assistant. In addition, patients will be asked to complete the questionnaires relating to disability (DASH score including optional modules), pain (VAS), health-related quality of life (SF-36, EQ-5D), health care consumption and production loss. From six weeks onwards, the research coordinator or research assistant will determine the Constant score.

At each clinical follow-up visit, anterior-posterior and lateral radiographs are generally routinely obtained. All images available from three months onwards will be analyzed. Apart for the 6-month follow-up, during which X-rays are needed for assessing signs of nonunion, local radiographical protocols will apply. For this reason, the follow-up at six month should not be done earlier. In case no radiographic healing is seen at six months, an X-ray at 12 months is also required. At the last visit, the surgeon or researcher will also document any secondary intervention that is planned for the patient.

### Sample size calculation

Calculation of the required sample size for the primary analysis is based on the assumption that the mean DASH in the non-operative group will be 16, with a Standard Deviation (SD) of 16 [8]. We expect less disability (*i.e.*, lower DASH score) at three months in the operative group; the expected DASH score in the operative group will be 10 (SD 10) [8]. A two-sided test with an α level of 0.05 and a β level of 0.2 requires 78 patients in every group. In order to account for loss of patients due to mortality (10%) and loss-to-FU (10% anticipated based upon previous studies by the research team), a sample size of 95 patients per group is needed.

Results of a retrospective study assessing clinical outcome of humeral shaft fractures, showed that 45-55% of all AO-subclasses were treated operatively [18]. In order to assess whether functional outcome scores differ between the fracture subtypes, a minimum of 2x20 patients per fracture subtype is sufficient. In order to achieve that, we need to include until at least 200 patients in both the operative groups and the non-operative group.

For the secondary analysis, some patients may be lost during the propensity score matching. Although we do not have a-priori data to determine how many patients will be lost, the 400 targeted patients will be more than sufficient.

### Statistical analysis

Data will be analyzed using the Statistical Package for the Social Sciences (SPSS) version 21 or higher (SPSS, Chicago, Ill., USA) and will be reported following the STrengthening the Reporting of OBservational studies in Epidemiology (STROBE) guidelines. Normality of continuous data will be assessed by inspecting the frequency distributions (histograms), and homogeneity of variances will be tested using the Levene’s test.

Descriptive analysis will be performed to report baseline characteristics (intrinsic variables and injury-related variables) and outcome measures for both treatment groups.

For continuous data mean and SD (parametric data) or medians and percentiles (non-parametric data) will be calculated and reported. For categorical data, numbers and frequencies will be calculated and reported for both treatment groups.

Univariate analysis will be performed in order to test the difference in the primary and secondary outcome measures between the operative and the non-operative group. Continuous data such as the DASH score at the different time points (primary outcome) will be tested using a Student’s T-test (parametric data) or a Mann Whitney U-test (non-parametric data). Chi-square analysis will be used for statistical testing of categorical data such as the nonunion rate. A p-value <0.05 will be taken as threshold of statistical significance.

For the primary analysis, a mixed linear regression model will be developed in order to model the relation between different covariates and the DASH score over time. Intrinsic and fracture-related variables that display a p-value <0.5 in univariate analyses will be added as covariate. Similar models will be developed for the Constant, SF-36, and EQ-5D score. Subgroup analysis (*e.g.*, elderly versus <65 years) will be performed.

For the secondary analysis we will develop a propensity score model as published before [43,44]. Characteristics including fracture type, age, gender, mechanism of injury, dominance, and activity levels will be included in this model; the resulting propensity score represents the chance of being operated. Next, the logit of the propensity score will be used in order to match each patient receiving operative treatment with one or more patients receiving non-operative treatment. The effect of operative treatment will be analyzed with linear or ordinal logistic mixed effects regression analysis taking the matched-pairs design into account. In the matched cohort, comparisons will be performed using a McNemar test (for categorical data), and a paired sample t test (parametric, continuous data) or a Wilcoxon signed rank test.

The economic evaluation will be performed from a societal perspective. Costs will be measured in accordance with Dutch guidelines for economic evaluations, using standard cost prices as published by Hakkaart-Van Rooijen et al. where possible [45]; effects will be discounted at a rate of 1.5% and costs at 4% per year [45]. The incremental cost-effectiveness ratio (ICER) of operative versus non-operative treatment will be expressed in a cost-utility ratio (*i.e.*, cost per QALY) using the EQ-5D utility score as effect measure. Uncertainty around this ratio will be presented using confidence ellipses on the cost-effectiveness plane and acceptability curves.

### Ethical considerations

The study will be conducted according to the principles of the Declaration of Helsinki (59th World Medical Association General Assembly, Seoul, October 2008). This study has been given a waiver of consent by the medical research ethics committee (MREC); in Dutch: Medisch Ethische Toetsings Commissie (METC). Following review of the protocol, the MREC concluded that this study is not subject to the Medical Research Involving Human Subjects Act (WMO). They concluded that the study is a medical/scientific research, but no patients are subjected to procedures or are required to follow rules of behavior. Consequently, the statutory obligation to provide insurance for subjects participating in medical research (article 7, Subsection 6 of the WMO and Medical Research (Human Subjects) Compulsory Insurance Decree of 23 June 2003) was also waived.

The MREC Erasmus MC (Rotterdam, The Netherlands) acts as central ethics committee for this trial (reference number MEC-2012-296). Approval has been obtained from the local hospital boards in all participating centers.

## Discussion

The HUMMER trial will study outcome after operative versus non-operative treatment of humeral shaft fractures. Operative treatment is expected to result in earlier recovery than non-operative treatment. Earlier functional recovery will result in a better quality of life of patients, earlier work and ADL resumption, a higher level of independency, and less health care needs. Although costs for initial treatment will be higher in the operative group (due to surgery), we hypothesize that costs will be saved by less health care needs during the recovery process and less productivity loss. Despite higher initial costs, we expect that primary surgery will be more cost-effective. To the best of our knowledge, this is the first high-quality multicenter prospective observational study that will look at patient, medical and societal perspective in patients with a humeral shaft fracture.

Thirty hospitals in the Netherlands will participate. Inclusion of patients has started October 01, 2012 and the expectation is to include 10 patients per month. With a follow-up of one year the presentation of data will be expected in the beginning of 2016.

## Abbreviations

ADL: Activities of daily living; AO: Arbeitsgemeinschaft für Osteosynthesefragen (German for “Association for the Study of Internal Fixation”); ASA: American Society of Anesthesiologists; BP: Bodily pain; DASH: Disabilities of the arm, shoulder and hand score; ED: Emergency Department; EQ-5D: EuroQol-5D; GH: General Health perception; HR-QoL: Health-related quality of life; MCS: Mental component summary; MH: General mental health; MREC: Medical Research Ethics Committee; NTR: Netherlands Trial Registry (in Dutch: Nederlands Trial Register); PCS: Physical component summary; PF: Physical functioning; QALY: Quality-adjusted life years; QoL: Quality of life; RCT: Randomized controlled trial; RE: Role limitations due to emotional problems; ROM: Range of motion; RP: Role limitations due to physical health; SD: Standard deviation; SF: Social functioning; SF-36: Short form-36; SPSS: Statistical package for the Social Sciences; STROBE: Strengthening the reporting of observational studies in Epidemiology guidelines; VAS: Visual analog scale; VT: Vitality, energy, or fatigue.

## Competing interests

The authors declare that they have no competing interests.

## Authors’ contributions

KCM, DDH, and EMMVL developed the trial and drafted the manuscript. DDH will act as trial principal investigator. SP assisted in the design of the health care consumption questionnaire and will perform the health economic analyses. KCM and EMMVL will perform statistical analysis of the trial data. KCM, EMMVL, HWB, PKB, MWGAB, MMMB, JDH, ARD, BJD, MGE, JCG, RH, MJH, AJHK, KAK, PAL, SAGM, RO, MP, RWP, BJP, WHR, GRR, JBS, NMRS, AKFT, EJTTH, MVDE, FHWMVDH, FMVDL, PVDZ, JPVD, HPWVJ, EJMMV, JPAMV, MW, PW, WPZ, SP, MHJV and DDH will participate in patient inclusion and assessment. All authors have read and approved the final manuscript.

## Pre-publication history

The pre-publication history for this paper can be accessed here:

http://www.biomedcentral.com/1471-2474/15/39/prepub
